# The mu opioid receptor and the orphan receptor GPR151 contribute to social reward in the habenula

**DOI:** 10.1038/s41598-022-24395-z

**Published:** 2022-11-24

**Authors:** Florence Allain, Michelle Carter, Sylvie Dumas, Emmanuel Darcq, Brigitte L. Kieffer

**Affiliations:** 1 Department of Psychiatry, Douglas Hospital Research Center, McGill University, Montreal, Canada; 2 INSERM U1114, Centre de Recherche en Biomédecine de Strasbourg, Université de Strasbourg, 1 rue Eugène Boeckel, CS60026, 67084 Strasbourg Cedex, France; 3 Oramacell, 75006 Paris, France

**Keywords:** Molecular biology, Neuroscience

## Abstract

The mu opioid receptor (MOR) and the orphan GPR151 receptor are inhibitory G protein coupled receptors that are enriched in the habenula, a small brain region involved in aversion processing, addiction and mood disorders. While MOR expression in the brain is widespread, *GPR151* expression is restricted to the habenula. In a previous report, we created conditional *ChrnB4-*Cre × *Oprm1*^*fl/fl*^ (so-called B4MOR) mice, where MORs are deleted specifically in Chrnb4-positive neurons restricted to the habenula, and shown a role for these receptors in naloxone aversion. Here we characterized the implication of habenular MORs in social behaviors. B4MOR^−/−^ mice and B4MOR^+*/*+^ mice were compared in several social behavior measures, including the chronic social stress defeat (CSDS) paradigm, the social preference (SP) test and social conditioned place preference (sCPP). In the CSDS, B4MOR^−/−^ mice showed lower preference for the social target (unfamiliar mouse of a different strain) at baseline, providing a first indication of deficient social interactions in mice lacking habenular MORs. In the SP test, B4MOR^−/−^ mice further showed reduced sociability for an unfamiliar conspecific mouse. In the sCPP, B4MOR^−/−^ mice also showed impaired place preference for their previous familiar littermates after social isolation. We next created and tested *Gpr151*^*−/−*^ mice in the SP test, and also found reduced social preference compared to *Gpr151*^+*/*+^ mice. Altogether our results support the underexplored notion that the habenula regulates social behaviors. Also, our data suggest that the inhibitory habenular MOR and GPR151 receptors normally promote social reward, possibly by dampening the aversive habenula activity.

## Introduction

Social interactions (maternal-, pair-, peer-, conspecifics-bonds) can either have benefits or costs^1^ and include various characteristics like social interest, social aptitude, social isolation/exclusion or social reciprocity. Social interactions are crucial in individual’s health: while positive social interactions (social reward) improve health and well-being throughout life, negative social interactions (social pain) can lead to pathological situations like health-damaging behaviors, stress, depression or suicide^2,3^. Reduced social reward valuation and/or increased reactivity to social rejection can be associated with different psychopathologies including depression^4–6^.

Mu opioid receptors (MORs) contribute to both positive and negative social behaviors. Social play behavior is either increased or decreased by morphine or naloxone, respectively^7–10^, and these effects extend to local injections in the nucleus accumbens—a central reward structure^11^. This was interpreted as an increased/decreased rewarding value of social play induced by MOR activation/blockade, respectively^12^. Using positron emission tomography in healthy human volunteers, social rejection was found to activate the MOR system in mood- and motivation-regulating brain sites (e.g. ventral striatum^13^), and this activation did not reach significance in patients suffering from major depressive disorder (MDD^14^). MOR activation in the nucleus accumbens also correlated with motivation for social interactions in healthy controls but not in MDD patients^14^. Thus, these data emphasize the MOR system as an important integrator of social information and MOR manipulation could be used to model social psychopathologies in animals. Indeed, mice lacking MORs display social attachment deficits (juveniles^15,16^) and an autistic-like behavior (adults^17–20^). MORs therefore influence different aspects of social behaviors, however circuit mechanisms and brain sites other than the nucleus accumbens have been little explored.

MORs are enriched in the medial subnucleus of the habenula (MHb^21^), an epithalamic structure implicated in mood-related dysfunctions^22–24^. Animal models of depression lead to an increase in metabolic activity in both nuclei of the habenula (medial and lateral^25,26^) and the MHb has been shown to be implicated in fear-^27,28^, aversion-^29,30^, or anxiety-^28,31,32^ responses. Emerging evidence also suggests a role for the MHb in social behaviors. Mice lacking the β4-subunit of the nicotine acetylcholine receptor, which is expressed almost exclusively in the MHb^33^, showed deficits in both nicotinic- and non-nicotinic reinforcing responses^34,35^ and, intriguingly, also showed increased heart rate in response to social isolation^36^. Further, mice lacking the Ca^+^ activated Cl-channel TMEM16A showed altered levels of anxiety/fear responses and social interactions^31^. Thus, concurrent with aversion processing, the MHb seems to modulate social behaviors. Whether the highly abundant MORs of the MHb are involved in social behaviors, has not been tested.

Recently we deleted MORs in ChrnB4-positive neurons (B4MOR^−/−^ mice), leading to a 50% reduction of total habenular MORs. We found that mutant mice are less sensitive to naloxone aversion without evident reward processing deficiencies^29^, suggesting that MORs (inhibitory receptors) may act as a brake to reduce the well-established aversive activity of habenula. Here we investigated whether MOR activity at this brain site also influences social responses. To do so, we evaluated sociability behaviors in B4MOR mice and our results show reduced response in all the tests. Because the MOR is an inhibitory G protein coupled receptor (GPCR), we also tested whether another inhibitory GPCR would play a similar role. We found social deficits in mice lacking the orphan GPCR GPR151, which is almost exclusively expressed in the MHb^37–41^. Altogether our data demonstrate for the first time that habenular receptors MOR and GPR151 contribute to facilitate social behaviors and suggest that the tonic control of MHb activity by inhibitory GPCRs is essential to maintain healthy social interactions.

## Materials and methods

### Mice

*Chrnβ4*-Cre mice^33^ were originally crossed with *Oprm1*^*fl/fl*^ mice^42^ to obtain *ChrnB4-*Cre ×* Oprm1*^*fl/fl*^ (abbreviated as B4MOR) mice lacking MORs in nicotinic acetylcholine receptor beta4 subunit-expressing neurons, primarily expressed in the medial habenula. The genetic mouse line was described earlier by our laboratory and maintained (at least ten generations) on a c57bl6:sv129 (50:50) background to produce experimental B4MOR^−/−^ mice and their B4MOR^+/+^ littermates as in^29,43^. Homozygous *Oprm1*^*−/−*^ mice (lacking MORs globally^44^) were created in our laboratory and are available at Jackson laboratories (B6.129S2-Oprm1^tm1Kff^/J Strain #:00755). *Gpr151*^*−/−*^ mice (c57bl6 background) were created using a knock-in strategy (Institut Clinique de la Souris, PHENOMIN, http://www.phenomin.fr), so that the fluorescent reporter eGFP is produced instead of the GPR151 receptor in homozygous null mutants (Fig. 4A, and see Suppl Methods for details on the construction and validation).

Commercial c57bl6:sv129 mice (Jackson Laboratories) were used as interactors for B4MOR^+/+^ and B4MOR^−/−^ mice in social preference and in the control procedure that paralleled the chronic social defeat stress procedure. CD-1 mice (Retired breeders, Charles River) were used as aggressors for the defeat. Commercial c57bl6:sv129 mice were used to judge the atypical behavior of B4MOR^−/−^ versus B4MOR^+/+^ mice, as well as *Oprm1*^−/−^ versus *Oprm1*^+/+^ mice in the reverse three chamber social test. Commercial c57bl/6J mice (Charles River) were used as interactors for *Gpr151*^*−/−*^ and *Gpr151*^+*/*+^ mice in the social preference test.

All procedures in this report were conducted in accordance with ARRIVE guidelines, the guidelines set forth by the Canadian Council of Animal Care and by the Animal Care Committees of McGill University/Douglas Mental Health University Institute and were also approved by the Regional Committee of Ethic in Animals Experiment of Strasbourg (CREMEAS, APAFIS#31880-2021072316283769 v1).

### Behavior

Descriptions of housing and behavioral procedures are in the Suppl Methods. This includes the description of chronic social defeat stress (CSDS) followed by a social interaction test (SIT), the social preference (SP) test, the social conditioned place preference test (sCPP), the food pellets self-administration paradigm, the reverse three chamber social test and the real time place preference testing.

### Statistics

Data are presented as means ± SEM. Social behavior tests (SIT after CSDS, SP test, reverse SP test, sCPP) were represented across time (habituation/pre-test versus social) and were analyzed using RM ANOVAs. Significant main effects or interaction effects (*P*’s ≤ 0.05) were followed by Holm-sidak’s multiple comparisons tests. Refer to Suppl Table 1 for description of all statistical tests.

## Results

### Both control and defeated B4MOR^−/−^ mice show reduced sociability to an unfamiliar CD1 mouse

**Figure 1:**
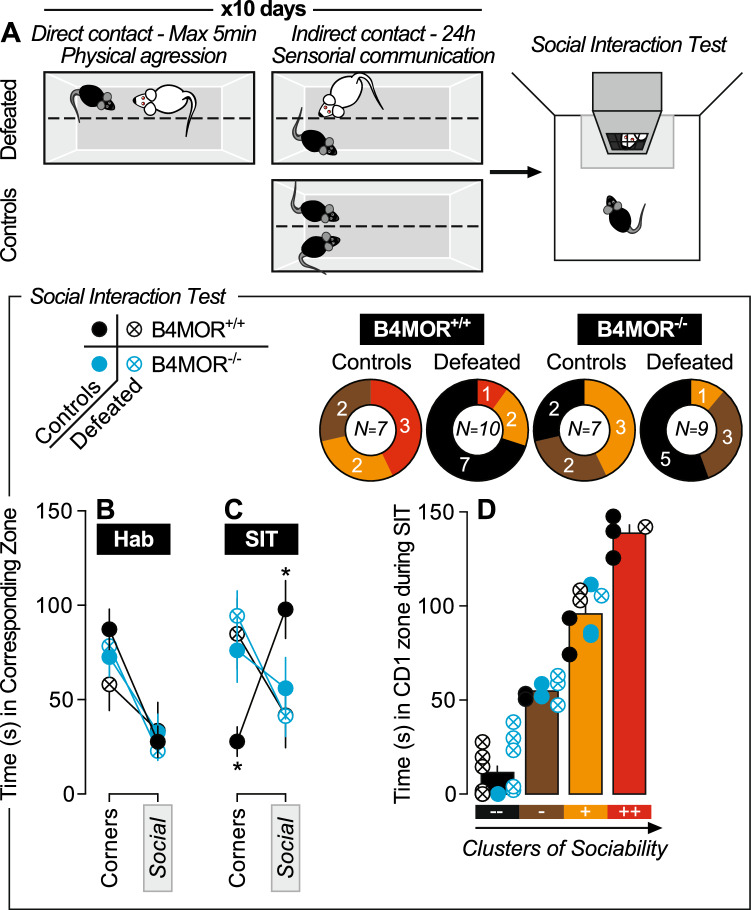
B4MOR^−/−^ mice show disinterest for a CD1-social target in the chronic social defeat paradigm. (**A**) Schematic representation of the chronic social defeat and control procedures followed by the social interaction test (the gray part represents the social interaction zone). The wire mesh enclosure was empty during the habituation of the test and contained an unfamiliar CD1 mouse during the social interaction test. (**B**) During habituation, mice spent generally more time in the corners than in the zone around the wire mesh enclosure (future social zone). (**C**) During the social interaction test, B4MOR^+/+^ control (undefeated) mice spent more time in the social zone and less time in the corners than B4MOR^+/+^ defeated mice. (**D**) Mice were clustered in four distinct categories based on their time spent in the social zone during the social interaction test. The less sociable cluster did not include B4MOR^+/+^ control mice, but all the three other groups were represented. *P’s < 0.05, B4MOR^+/+^ control mice versus B4MOR^+/+^ defeated mice. N’s = 7–10/group.

Because the habenula is a notorious aversion center and MOR presumably inhibits the aversive habenula activity^29,45^, we first investigated the hypothesis that chronic social stress (social defeat) would trigger enhanced social avoidance in B4MOR^−/−^ mice. We tested B4MOR^−/−^ and B4MOR^+/+^ mice in a chronic social defeat stress paradigm^46^ for 10 consecutive days. Control mice were not exposed to physical aggressions. Twenty-four hours after the procedure, sociability for a novel CD1 mouse was tested in an open field (2.5-min habitation period followed by 2.5-min social interaction test; Fig. 1A).

We found that the time spent in the CD1/social and corners/non-social zones depended upon the phase of the test (habituation versus social test), the previous social stress exposure, and the genotype (Fig. 1B,C). During habituation, where the wire mesh enclosure was empty, both B4MOR^−/−^ and B4MOR^+/+^ mice spent significantly more time in the corners compared to the CD1 zone independently of the previous social stress experience (Stress x Zone interaction effect, *P*’s > 0.05; Fig. 1B), suggesting a general preference for corners in our experimental set up. However, during the social interaction test (SIT), where a novel CD1 mouse was inserted into the wire mesh enclosure, previous social stress exposure altered the time spent in the CD1 zone versus corners only in B4MOR^+/+^ mice (Stress x Zone interaction effect, F_1,15_ = 6.55, *p* = 0.02; Fig. 1C). Thus, as expected, defeated B4MOR^+/+^ spent more time in the corners and less in the social zone compared to control (undefeated) B4MOR^+/+^ mice (*P*’s < 0.05; Fig. 1C). On the contrary, both B4MOR^−/−^ controls and defeated mice spent more time in corners, whether a CD1 mouse was present or not in the wire mesh enclosure, suggesting that basal sociability is reduced in the mutant mice.

We then clustered individual mice during the social interaction (time spent in the CD1 zone) based on their sociability index using the WARD method (Fig. S1). We found four sociability clusters, distinguishing resilient and susceptible subcategories of mice^46,47^ (Fig. 1D). There was a significant association between the four clusters and the stress/genotype groups (*χ*^*2*^(9) = 17.75, *p* = 0.04; Fig. 1D) which was driven by the undefeated B4MOR^+/+^ controls: removing this group from the analysis made the association non-significant (*χ*^*2*^(6) = 7.88,* p* = 0.25; Fig. 1D). This suggests that mice avoiding the social zone are equally distributed in defeated groups from both genotypes, but also in control (undefeated) B4MOR^−/−^ mice—which was intriguing as this group was previously not exposed to chronic social stress. This finding is another indication that B4MOR^−/−^ mice display reduced sociability at baseline.

### B4MOR^−/−^ mice show reduced social preference for a partner of the same strain

**Figure 2:**
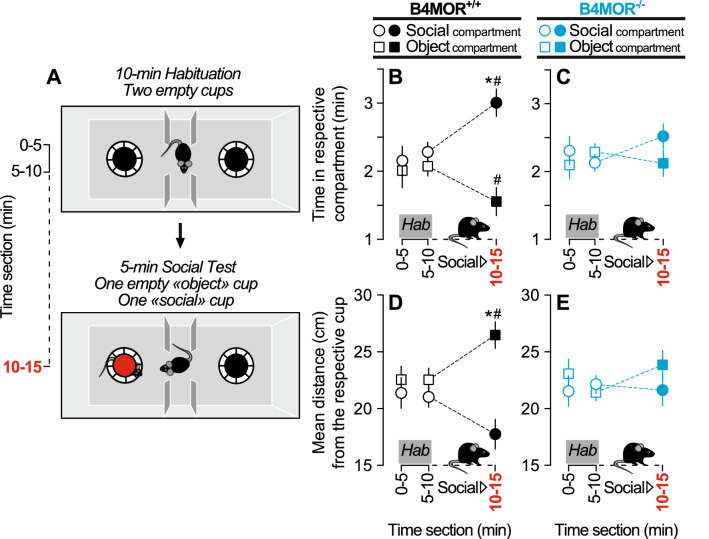
B4MOR^−/−^ mice do not express social preference in the three-chamber social preference test. (**A**) Schematic representation of the social preference test. (**B,C**) Time spent in the social versus object compartment in B4MOR^+/+^ and B4MOR^−/−^ mice, respectively. (**D,E**) Distance from the social versus object cup in B4MOR^+/+^ and B4MOR^−/−^ mice, respectively. During the social test, B4MOR^+/+^ mice spent more time in the social compartment (**B**) and were closer to the social cup (**D**). B4MOR^−/−^ mice spent a similar amount of time in both compartments (**C**) and were at equidistance from the two cups (**E**). *P’s < 0.05, Object compartment versus social compartment. ^#^*P*’s < 0.05, habituation (Hab) versus social test. N’s = 21–24/group.

To further investigate a potential deficit of social interest in B4MOR^−/−^ mice, we compared B4MOR^−/−^ with B4MOR^+/+^ mice in the three-chamber social test, to assess social preference for an unfamiliar mouse. In this test, mice have free choice to spend time in the three chambers of the apparatus during a 10-min habituation period followed by a 5-min social test (Fig. 2A). During habituation, empty cups are placed in the two opposite chambers. Both B4MOR^−/−^ and B4MOR^+/+^ mice similarly explored the two chambers during habituation (Fig. 2B,C). Then, an unfamiliar mouse was placed under one of the two cups and we compared the last 5-min bin of the habituation phase (5–10 min) with the 5-min social test (10–15 min). The time spent in the social versus object chamber depended upon the test phase only in B4MOR^+/+^ mice (Test x Chamber interaction effect, B4MOR^+/+^, F_1,20_ = 12.55, *p* = 0.002; Fig. 2B; B4MOR^−/−^, F_1,23_ = 2.46, *p* = 0.13; Fig. 2C). As expected, B4MOR^+/+^ mice spent more time in the social-paired chamber compared to the non-social one (*p* < 0.0001; Fig. 2B) leading them to be closer to the social cup versus the empty/object cup (*p* < 0.0001; Fig. 2D). However, B4MOR^−/−^ mice spent a similar amount of time in the two chambers (Fig. 2C) leading them to be at equidistance between the two cups (Fig. 2E). Added to our previous results, these data suggest a social preference deficit in B4MOR^−/−^ mice.

### B4MOR^−/−^ mice do not show conditioned place preference to social interactions

**Figure 3:**
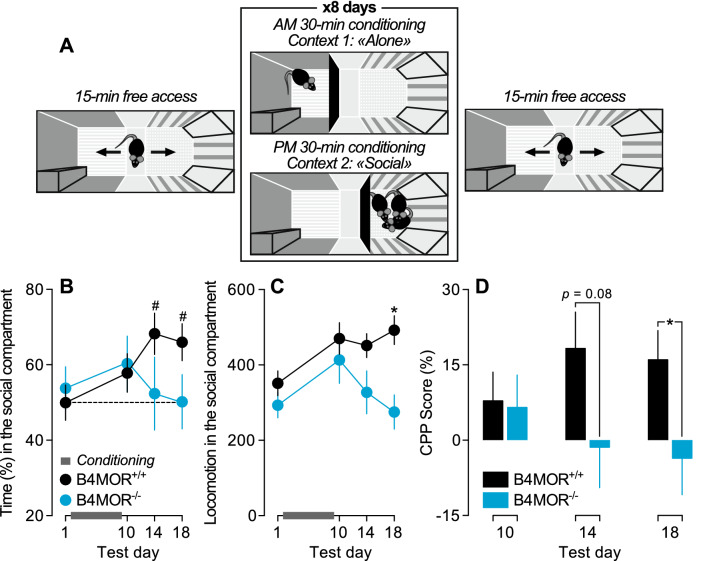
B4MOR^−/−^ mice do not show conditioned place preference to social interactions. (**A**) shows a schematic representation of the social conditioned place preference paradigm. (**B**) The time spent in the social-paired compartment started at 50% in both genotypes on test day 1 and increased after social conditioning only in B4MOR^+/+^ mice. (**C**) Locomotor activity (beam breaks and release) in the social-paired compartment was higher in B4MOR^+/+^ mice versus B4MOR^−/−^ mice after social conditioning. (**D**) CPP score was higher in B4MOR^+/+^ mice versus B4MOR^−/−^ mice on test day 18. ^#^*P*’s < 0.05, versus Test Day 1. *P’s < 0.05, B4MOR^+/+^ versus B4MOR^−/−^ mice. N’s = 13–15/group.

We next investigated the social reward-context association using a social Conditioned Place Preference (CPP) paradigm in B4MOR^−/−^ and B4MOR^+/+^ mice (Fig. 3A). Reward-context association is an indicator of reward valuation of a conditioned stimulus^48^, in our case social stimuli. After a pre-test where mice could freely explore a CPP apparatus (50%–50% of time spent in the allocated social versus non-social compartment, Test Day 1, Fig. 3A,B), mice were socially isolated and were next conditioned to one compartment alone every morning and with their ex-littermates in the other compartment every afternoon for 8 successive days (Fig. 3A). While repeated social exposure to ex-littermates conditioned a place preference in B4MOR^+/+^ mice, this was not the case for B4MOR^−/−^ mice (Genotype x Test Day interaction effect, F_3,78_ = 2.86, *p* = 0.04; versus Test Day 1, *P’s* < 0.05 at Test Days 14 and 18 in B4MOR^+/+^ mice; All other *P*’s > 0.05; Fig. 3B). Locomotor activity in the social compartment was also dependent upon previous social conditioning and genotype (Main effect of Test Day, F_3,78_ = 4.88, *p* = 0.004; Main effect of Genotype, F_1,26_ = 5.72, *p* = 0.02; Genotype x Test Day interaction effect, F_3,78_ = 2.92, *p* = 0.04; Fig. 3C) leading to decreased locomotor activity in the social-paired compartment on the last test day in B4MOR^−/−^ mice versus B4MOR^+/+^ mice (*p* = 0.002, Fig. 3C). The CPP score was significantly higher in B4MOR^+/+^ mice on test day 18 (*p* = 0.04; Fig. 3D) but not on test days 10 (*p* = 0.88; Fig. 3D) and 14 (*p* = 0.08; Fig. 3D). Altogether, these results suggest that social reward-context association occurs only in B4MOR^+/+^ mice and that social reward is lower in B4MOR^−/−^ mice compared to B4MOR^+/+^ mice.

In this test, B4MOR^−/−^ mice deficits to value social interactions as rewarding could be the consequence of learning and/or memory deficits. We then tested whether low reward valuation also applied to palatable food. B4MOR^−/−^ and B4MOR^+/+^ mice self-administered similar amounts of chocolate pellets under FR1, FR5 and PR (Fig. S2) suggesting that food reinforcement was not altered in B4MOR^−/−^ mice. When tested under reversal conditions (active and inactive ports were switched), the flexibility to learn the new task was similar for B4MOR^−/−^ and B4MOR^+/+^ mice. Of note, our previous work showed that B4MOR^−/−^ mice also show intact Morphine CPP^29^. Altogether, these data suggest that no generalized learning and/or memory deficit seems to impair the social reward-context association in B4MOR^−/−^ mice.

### *Gpr151−/−* mice also show reduced social preference for a partner of the same strain

**Figure 4:**
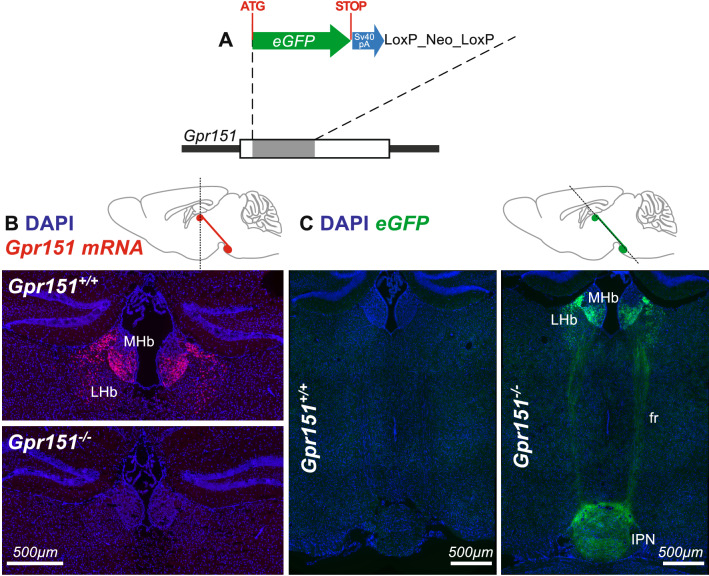
Generation and characterization of *Gpr151*^*−/−*^ knock-in mice. (**A**) Construction of the *Gpr151*^*−/−*^ knock-in mouse line. (**B**) *Gpr151* mRNA in *Gpr151*^+*/*+^ and *Gpr151*^*−/−*^ brain slices using FISH. (**C**) eGFP signal detected by immunohistochemistry in a 50°–60° coronal brain section of a *Gpr151*^*−/−*^ mouse throughout the habenular pathway: medial habenula (MHb), lateral habenula (LHb), fasciculus retroflexus (fr) and interpeduncular nucleus (IPN).

To extend our study to another inhibitory habenular receptor, we used a genetic mouse line where the GPR151 receptor was removed. The construction of this mouse line was designed as to show *eGFP* expression upon deletion of the *Gpr151* gene coding sequence (Fig. 4A). Analysis of the *Gpr151* transcript in coronal brain slices of *Gpr151*^*−/−*^ mice versus *Gpr151*^+*/*+^ mice by sFISH (see Suppl Methods) confirmed the absence of *Gpr151* mRNA in the habenular complex of *Gpr151*^*−/−*^ mice (Fig. 4B). Also, sectioning coronal brain slices of *Gpr151*^*−/−*^ mice with a 50°–60° angle allowed us to visualize eGFP*-*positive cells in which the GPR151 receptors were removed. As expected, the eGFP fluorescence was detected throughout the habenulo-interpeduncular pathway in *Gpr151*^*−/−*^ mice but not *Gpr151*^+*/*+^ mice (Fig. 4C). There was almost no eGFP expression in the dorsal part of the medial habenula (Fig. 4C), which agrees with results reporting no detection of GPR151 in Substance P-expressing cells^38^.

**Figure 5:**
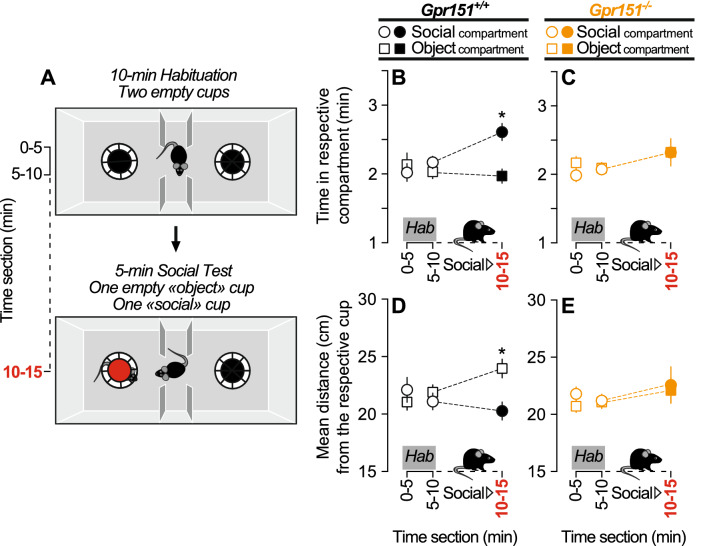
*Gpr151*^*−/−*^ mice do not express social preference in the three-chamber social preference test. (**A**) Schematic representation of the social preference test. (**B,C**) Time spent in the social versus object compartment in *Gpr151*^+*/*+^ and *Gpr151*^*−/−*^ mice, respectively. (**D,E**) Distance from the social versus object cup in *Gpr151*^+*/*+^ and *Gpr151*^*−/−*^ mice, respectively. During the social test, *Gpr151*^+*/*+^ mice spent more time in the social compartment (**B**) and were closer to the social cup (**D**). *Gpr151*^*−/−*^ mice spent a similar amount of time in both compartments (**C**) and were at equidistance from the two cups (**E**). *P’s < 0.05, object compartment versus social compartment. N’s = 19–20/group.

Because MOR and GPR151 are both inhibitory habenular receptors, we hypothesized that the social reward deficiencies measured in B4MOR^−/−^ mice could also be measured in *Gpr151*^*−/−*^ mice. We assessed social preference for an unfamiliar mouse of the same strain in *Gpr151*^*−/−*^ mice and *Gpr151*^+*/*+^ mice using the three-chamber social test (Fig. 5A). For both genotypes, there was no discrimination between the two opposite chambers during habituation (Fig. 5B,C). During the subsequent social test, the preference for the social-paired versus non-social paired chamber occurred in *Gpr151*^+*/*+^ (*p* = 0.028, Fig. 5B) but not in *Gpr151*^*−/−*^ mice (Fig. 5C, also see Fig. S3 for data in female mice). *Gpr151*^+*/*+^ mice were then closer to the social cup (*p* = 0.048, Fig. 5D) while *Gpr151*^*−/−*^ stayed at equidistance from the two cups (Fig. 5E). These data suggest that the social reward deficit observed in B4MOR^−/−^ mice could extend to other models of mice lacking inhibitory habenular receptors.

## Discussion

In this report, we first show that mice lacking *MOR*s in the MHb present reduced sociability in the chronic social defeat paradigm at baseline (no stress), suggesting a social interaction deficit. We next show that these mutant mice show reduced sociability in the three-chamber social preference test, as well as reduced place preference to a social context, confirming the alteration of social behaviors. We finally show reduced sociability in the social preference test in mice lacking GPR151, an orphan GPCR enriched in the MHb. Altogether, these data support a role for Gi/o coupled GPCRs of the MHb in promoting social behaviors, likely through the tonic inhibition of MHb activity.

### The MHb regulates social behaviors

Only few studies have proposed a role of the MHb in social behaviors^31,36^. Indeed, habenula function is mainly studied in the context of mood disorders. Habenular activity was reported to be increased in MDD patients^49^. Consistent with this finding, animal models of depression show increased metabolic activity in both the medial (MHb) and the lateral (LHb) nuclei of the habenula while neural activity is generally lowered in other brain areas^25,26^. The function of the LHb in depressive-like states has been well characterized^50^ and reduced LHb activity parallels reduced depressive-like symptoms^51,52^. Neural activity in the MHb and the interpeduncular nucleus (IPN)—its main projection site—is also increased in animal models of depression^25^. MORs have their strongest expression in the MHb^21^ and the MOR system is already known to regulate depressive-like states^53,54^. We therefore here evaluated the role of habenular MORs^29^ using a model of depression, the chronic social defeat paradigm^55,56^, with the assumption that MOR activity may alleviate the aversive consequences of stress, and limit social avoidance in this test. Unexpectedly, while no genotype effect was observed in the stress response, mice lacking habenular MORs showed a social deficit at baseline. This was a first indication for a role of the MHb in the regulation of social behaviors, which we further confirmed through several social testing paradigms and the genetic deletion of another inhibitory GPCR specific to the MHb.

### MORs promote social behaviors at the level of the MHb

The implication of MORs in reward- and pain-related processes is well characterized^57,58^, and was also shown to extend to the social aspects of behavior^3^. In the literature, the alteration of *MOR* function was measured in different mental health disorders with social deficiencies, including depression^14,53,59–61^. Thus, constitutive MOR knockout mice (*Oprm1*^−/−^ mice) present a large range of social deficiencies, including a decrease in social attachment, reciprocal direct social interactions, social preference, social conditioned place preference, ultrasound vocalizations (USVs) emission in response to social cues and social exploratory activation to USVs^15–20^. The pharmacology also indicates that MOR activation/blockade increases/decreases social responses, respectively^7–12^. MOR activity at the level of the mesolimbic pathway partly contributes to these effects^11^ but nothing is known at the level of the habenula, where MOR density is extremely high^21^.

In this study, mice lacking MORs in the MHb show reduced sociability (or propensity to spend time with another mouse rather than an empty chamber, see^62^) in two different tasks. In the first task (chronic social defeat), undefeated B4MOR^−/−^ mice behaved like defeated B4MOR^+/+^ mice with a reduced social exploration (Fig. 1), suggesting that in the absence of social stress, at baseline levels, habenular MORs facilitate sociability to congeners with different phenotypic traits. In this procedure however, mice were isolated for 10 days, which could induce a social distress by itself possibly recruiting the opioid system^7,63^ and be sufficient to impair future sociability in mutant mice. In the second sociability task (three-chamber social preference), mice were group housed and social preference for an unfamiliar peer from the same strain was tested. Under these different conditions, B4MOR^−/−^ mice did not dissociate between the social and the empty compartment (Fig. 2), characterizing them as not sociable or indifferent to the social target. Together, the two testing procedures concur to demonstrate that MORs operate at the level of the MHb to promote sociability. Whether this mechanism acts in concert with the mesolimbic circuitry, or perhaps the dorsal raphe strongly involved in social behaviors^64,65^, remains to be clarified.

### MORs in the MHb encode the hedonic value of social stimuli

We previously highlighted a role of habenular MORs in aversive states^29,45^ and using Mor-Cre mice^66^, we recently showed that the selective activation of Hb-IPN MORs positive cells was aversive^45^. Thus, MORs function could balance between reward and aversion processes at the level at the habenula. Decreased sociability in B4MOR^−/−^ mice could be the consequence of both processes, either an increase in social aversion or a decrease in social reward. Our results tend to the latter hypothesis as social stimuli-context association did not occur in B4MOR^−/−^ mice—without the expression of an avoidance to the social-context (Fig. 3). This suggests that the hedonic value of social interactions with siblings is reduced in mice lacking habenular MORs.

It has recently been shown that mice lacking MORs constitutively demonstrate an “atypical” social behavior leading judge mice to decrease their interaction time with them^18^. We did not find this effect in mice lacking MORs in the MHb (Fig. S4), suggesting that an “atypical” behavior does not explain decreased sociability observed in B4MOR^−/−^ mice. Added to this result, social reward-context association occurred in both B4MOR^+/+^ mice conditioned only with B4MOR^+/+^ and with mixed genotypes (data not shown). Thus, abnormal/atypical social behavior does not seem to contribute to deficits in social interactions behavior in B4MOR^−/−^ as it is likely the case for constitutive *Oprm1*^*−/−*^ mice^17,18,61^.

### GPR151, as MORs in the MHb, facilitates sociability

Our results on the implication of MHb MORs in social behavior led us to question the role of other inhibitory habenular receptors in social interactions. GPR151 is an orphan GPCR densely and almost exclusively expressed in the habenular pathway^37–41^, and this highly restricted expression pattern in the brain makes this particular GPCR a highly valuable potential target for brain disease. Little is known about the function of GPR151, but an implication of GPR151 in nicotine intake was shown, supposing a role for this receptor in drug addiction and reward processes^40^. We hypothesized that, as for MHb MORs, GPR151 may act as a brake on the habenulo-interpeduncular pathway. The mechanism by which these receptors would be endogenously active is still to be determined, but if this is the case, we would expect *Gpr151*^*−/−*^ mice to show impaired sociability. Indeed, while *Gpr151*^+*/*+^ mice showed sociability for a peer (three-chamber social preference), *Gpr151*^*−/−*^ mice were indifferent to a social partner (Fig. 5). This result extends our data from B4MOR mice and supports the more general notion that deleting inhibitory GPCRs from the MHb reduces social behaviors, likely via enhanced activity of the habenular pathway. Of note, mice are nocturnal animals but were tested during the light phase which could have impacted sociability. However, social interaction behavior can be evaluated during both the dark and the light phase of the circadian cycle and there is no clear influence of the testing time^67^.

As habenular function is related to anxiety responses^28,31,32^, we can hypothesize that the observed decrease in sociability could result from changes in anxiety levels. However, we found that B4MOR^−/−^ and B4MOR^+/+^ mice similarly explored the center of an open field apparatus (Fig. S5) and Antolin-Fontes et al.^40^, reported that *Gpr151*^*−/−*^ mice spend similar amount of time in the open arms of an elevated plus maze apparatus compared to *Gpr151*^+*/*+^ mice. Further, the latency to first entry in the social compartment was not impaired in B4MOR and GPR151 null mutants (Fig. S6), suggesting altogether that mutant mice are not less sociable because of higher anxiety.

## Conclusion

Altogether, our results suggest that habenular Gi/o receptors participate to the attribution of the hedonic value of social interactions. Whether this function is related to hedonic or motivational aspects of social interactions is unknown and could be determined using operant social self-administration models^68^. It is interesting to note that double in situ hybridization allowed us to observe habenular cells expressing MOR only, GPR151 only or both receptors (Fig. S7). Thus, it will be interesting in the future to quantify the proportion of habenular cells expressing both receptors and eventually address the question of potential interactions between the two receptors in the MHb. Also it will be important to precisely characterize cells of the MHb responsible for the regulation of social behaviors as MOR- and GPR151-positive neurons overlap cholinergic neurons known to populate the MHb^21,38^, as well as Substance P neurons (*MOR*, see^21^). Finally, a next step could be the identification of circuit mechanisms underlying the roles of habenular MOR and GPR151 in the regulation of social behaviors. We recently showed that optostimulation of habenular MOR-positive neurons projecting to the IPN is aversive^45^, an activity that could partly contribute to the impaired sociability observed in mice lacking habenular MORs. However similar optostimulation parameters applied to habenular GPR151-positive neurons projecting to the IPN did not seem to be aversive (Fig. S8). Although this finding requires further confirmation, it is possible that distinct cellular and/or circuit mechanisms underlie the apparent similar role of MORs and GPR151 receptors in regulating social behaviors, and these remain to be determined.

In summary, our findings reveal the importance of MHb activity, and receptors modulating this activity, in the control of social behaviors. This study opens new therapeutic perspectives, because social interactions deficits are associated with several psychopathologies including addiction^69^ and depression^3^, therefore targeting habenula function may benefit to this particular dimension of psychiatric conditions. In particular, the development of *GPR151* agonists may deliver highly specific drugs with minimal adverse effects, in high demand in the area of mental health.

## Supplementary Information

Supplementary Table 1.

Supplementary Figures.

Supplementary Information.

## Data Availability

The datasets used and/or analyzed during the current study are available from the corresponding author on reasonable request.
