# Supplementary material for: The mu opioid receptor and the orphan receptor GPR151 contribute to social reward in the habenula
Source: Sci Rep. 2022 Nov 24;12:20234. doi: 10.1038/s41598-022-24395-z (PMC9691715; doi:10.1038/s41598-022-24395-z)
Supplement: Supplementary file 1 — Supplementary Table 1. [file 41598_2022_24395_MOESM1_ESM.pdf]

MAIN FIGURES			TEST	MAIN EFFECTS<br>P's < 0.05; P's < 0.1 (*P's and #P's are reported on respective figures)	HOLM-SIDAK'S multiple comparisons tests
Figure 1	1B-C	B4MOR$^{+/+}$ versus B4MOR$^{-/-}$	4-way ANOVA: Genotype x Stress x Test x Zone, the latter two as RM variables<br>- Genotype: B4MOR$^{+/+}$ versus B4MOR$^{-/-}$<br>- Stress: Controls versus Defeated<br>- Test: Habituation versus SIT<br>- Zone: CD1 Zone versus Corners	- Genotype x Stress x Test x Zone interaction effect, $F_{1, 29} = 5.05$, $p = 0.03$	
Figure 1	1B	B4MOR$^{+/+}$	*Habituation (Hab)*<br>2-way ANOVA: Sress x Zone, the latter as a RM variable<br>- Stress: Controls versus Defeated<br>- Zone: CD1 Zone versus Corners	- Main effect of Stress, $F_{1, 15} = 1.75$, $p = 0.21$<br>- Main effect of Zone, $F_{1, 15} = 6.18$, $p = 0.03$<br>- Stress x Zone interaction effect, $F_{1, 15} = 1.08$, $p = 0.31$	
Figure 1	1B	B4MOR$^{-/-}$	*Habituation (Hab)*<br>2-way ANOVA: Sress x Zone, the latter as a RM variable<br>- Stress: Controls versus Defeated<br>- Zone: CD1 Zone versus Corners	- Main effect of Stress, $F_{1, 14} = 0.13$, $p = 0.72$<br>- Main effect of Zone, $F_{1, 14} = 29.59$, $p < 0.0001$<br>- Stress x Zone interaction effect, $F_{1, 14} = 0.81$, $p = 0.38$	
Figure 1	1C	B4MOR$^{+/+}$	*Social Interaction Test*<br>2-way ANOVA: Sress x Zone, the latter as a RM variable<br>- Stress: Controls versus Defeated<br>- Zone: CD1 Zone versus Corners	- Main effect of Stress, $F_{1, 15} = 0.003$, $p = 0.96$<br>- Main effect of Zone, $F_{1, 15} = 0.37$, $p = 0.55$<br>- Stress x Zone interaction effect, $F_{1, 15} = 6.55$, $p = 0.02$	- Controls versus Defeated: Corners, *$p = 0.047$; CD1 Zone, *$p = 0.047$.
Figure 1	1C	B4MOR$^{-/-}$	*Social Interaction Test*<br>2-way ANOVA: Sress x Zone, the latter as a RM variable<br>- Stress: Controls versus Defeated<br>- Zone: CD1 Zone versus Corners	- Main effect of Stress, $F_{1, 14} = 0.23$, $p = 0.64$<br>- Main effect of Zone, $F_{1, 14} = 3.47$, $p = 0.08$<br>- Stress x Zone interaction effect, $F_{1, 14} = 0.71$, $p = 0.41$	
Figure 1	1D	B4MOR$^{+/+}$ versus B4MOR$^{-/-}$	Chi-squared test<br>- Ward method (Time in CD1 zone, **Figure S1**)<br>- Groups: B4MOR$^{+/+}$ Controls, B4MOR$^{+/+}$ Defeated, B4MOR$^{-/-}$ Controls, B4MOR$^{-/-}$ Defeated	χ^2 (9) = 17.75, $p = 0.038$	
Figure 1	1D	B4MOR$^{+/+}$ versus B4MOR$^{-/-}$	Chi-squared test<br>- Ward method (Time in CD1 zone, **Figure S1**)<br>- Groups: B4MOR$^{+/+}$ Defeated, B4MOR$^{-/-}$ Controls, B4MOR$^{-/-}$ Defeated	χ^2 (6) = 7.88, $p = 0.247$	
Figure 2	2B	B4MOR$^{+/+}$	2-way ANOVAs: Test x Chamber, both as RM variables<br>- Test: 5-10min Hab bin versus 10-15min social bin<br>- Chamber: Object versus Social	- Main effect of Test, $F_{1, 20} = 3.54$, $p = 0.7$<br>- Main effect of Chamber, $F_{1, 20} = 7.93$, $p = 0.01$<br>- Test x Chamber interaction effect, $F_{1, 20} = 12.55$, $p = 0.002$	- Object versus social chamber: 5-10min, $p = 0.41$; 10-15min, *$p < 0.0001$<br>- Habituation versus social test: Object, #$p = 0.0497$; social, #$p = 0.02$
Figure 2	2C	B4MOR$^{-/-}$	2-way ANOVAs: Test x Chamber, both as RM variables<br>- Test: 5-10min Hab bin versus 10-15min social bin<br>- Chamber: Object versus Social	- Main effect of Test, $F_{1, 23} = 17.33$, $p = 0.0004$<br>- Main effect of Chamber, $F_{1, 23} = 0.22$, $p = 0.64$<br>- Test x Chamber interaction effect, $F_{1, 23} = 2.46$, $p = 0.13$	- Habituation versus social test: Object, $p = 0.51$; social, $p = 0.25$
Figure 2	2D	B4MOR$^{+/+}$	2-way ANOVAs: Test x Chamber, both as RM variables<br>- Test: 5-10min Hab bin versus 10-15min social bin<br>- Chamber: Object versus Social	- Main effect of Test, $F_{1, 20} = 1.36$, $p = 0.26$<br>- Main effect of Chamber, $F_{1, 20} = 7.53$, $p = 0.01$<br>- Test x Chamber interaction effect, $F_{1, 20} = 9.99$, $p = 0.005$	- Object versus social chamber: 5-10min, $p = 0.36$; 10-15min, *$p < 0.0001$<br>- Habituation versus social test : Object, #$p = 0.048$; social, $p = 0.056$
Figure 2	2E	B4MOR$^{-/-}$	2-way ANOVAs: Test x Chamber, both as RM variables<br>- Test: 5-10min Hab bin versus 10-15min social bin<br>- Chamber: Object versus Social	- Main effect of Test, $F_{1, 23} = 16.75$, $p = 0.0004$<br>- Main effect of Chamber, $F_{1, 23} = 0.18$, $p = 0.67$<br>- Test x Chamber interaction effect, $F_{1, 23} = 1.6$, $p = 0.22$	- Habituation versus social test: Object, $p = 0.28$; social, $p = 0.76$

**Figure 3**	**3B**	$B4MOR^{+/+}$ versus $B4MOR^{-/-}$	2-way ANOVA: Genotype x Test, the latter as a RM variable<br>- Genotype: $B4MOR^{+/+}$ versus $B4MOR^{-/-}$<br>- Test: Days 1, 10, 14 and 18	- Main effect of Genotype, $F_{1, 26} = 0.84$, $p = 0.37$<br>- Main effect of Test, $F_{3, 78} = 1.34$, $p = 0.27$<br>- Genotype x Test interaction effect, $F_{3, 78} = 2.86$, $p = 0.04$	- Versus test day 1:<br>$B4MOR^{+/+}$: Test 10, $p = 0.21$; Test 14, $^{\#}p = 0.01$; Test 18, $^{\#}p = 0.02$<br>$B4MOR^{-/-}$: Test 10, $p = 0.7$; Test 14, $p = 0.84$; Test 18, $p = 0.84$
	**3C**		2-way ANOVA: Genotype x Test, the latter as a RM variable<br>- Genotype: $B4MOR^{+/+}$ versus $B4MOR^{-/-}$<br>- Test: Days 1, 10, 14 and 18	- Main effect of Genotype, $F_{1, 26} = 5.72$, $p = 0.02$<br>- Main effect of Test, $F_{3, 78} = 4.88$, $p = 0.004$<br>- Genotype x Test interaction effect, $F_{3, 78} = 2.92$, $p = 0.04$	- $B4MOR^{+/+}$ versus $B4MOR^{-/-}$:<br>Test 1: $p = 0.57$; Test 10: $p = 0.57$; Test 14: $p = 0.13$; Test 18: $^{*}p = 0.002$
	**3D**		Unpaired t-tests: $B4MOR^{+/+}$ versus $B4MOR^{-/-}$	- Test 10: $t_{26} = 0.16$, $p = 0.88$<br>- Test 14: $t_{26} = 1.82$, $p = 0.08$<br>- Test 18: $t_{26} = 2.12$, $^{*}p = 0.04$	
**Figure 5**	**5B**	*Gpr151*$^{+/+}$	2-way ANOVA: Test x Chamber, both as RM variables<br>- Test: 5-10min Habituation bin versus 10-15min social bin<br>- Chamber: Object versus Social	- Main effect of Test, $F_{1, 19} = 49.27$, $p < 0.0001$<br>- Main effect of Chamber, $F_{1, 19} = 11.87$, $p = 0.003$<br>- Test x Chamber interaction effect, $F_{1, 19} = 2.26$, $p = 0.15$	- Object versus social chamber: 5-10min, $p = 0.57$; 10-15min, $^{*}p = 0.028$<br>- Habituation versus social test : Object, $p = 0.8$; social, $p = 0.15$
	**5C**	*Gpr151*$^{-/-}$	2-way ANOVA: Test x Chamber, both as RM variables<br>- Test: 5-10min Habituation bin versus 10-15min social bin<br>- Chamber: Object versus Social	- Main effect of Test, $F_{1, 18} = 30.13$, $p < 0.0001$<br>- Main effect of Chamber, $F_{1, 18} = 0.003$, $p = 0.96$<br>- Test x Chamber interaction effect, $F_{1, 18} = 0.002$, $p = 0.97$	- Habituation versus social test : Object, $p = 0.62$; social, $p = 0.62$
	**5D**	*Gpr151*$^{+/+}$	2-way ANOVA: Test x Chamber, both as RM variables<br>- Test: 5-10min Habituation bin versus 10-15min social bin<br>- Chamber: Object versus Social	- Main effect of Test, $F_{1, 19} = 20.62$, $p = 0.0002$<br>- Main effect of Chamber, $F_{1, 19} = 5.19$, $p = 0.035$<br>- Test x Chamber interaction effect, $F_{1, 19} = 1.83$, $p = 0.19$	- Object versus social chamber: 5-10min, $p = 0.6$; 10-15min, $^{*}p = 0.048$<br>- Habituation versus social test : Object, $p = 0.34$; social, $p = 0.59$
	**5E**	*Gpr151*$^{-/-}$	2-way ANOVA: Test x Chamber, both as RM variables<br>- Test: 5-10min Habituation bin versus 10-15min social bin<br>- Chamber: Object versus Social	- Main effect of Test, $F_{1, 18} = 10.19$, $p = 0.005$<br>- Main effect of Chamber, $F_{1, 18} = 0.04$, $p = 0.85$<br>- Test x Chamber interaction effect, $F_{1, 18} = 0.02$, $p = 0.9$	- Habituation versus social test : Object, $p = 0.72$; social, $p = 0.72$

SUPPLEMENTARY FIGURES			**TEST**	**MAIN EFFECTS**<br>*P*'s < 0.05; *P*'s < 0.1 (**P*'s and #*P*'s are reported on respective figures)	**HOLM-SIDAK'S multiple comparisons tests**
**Figure S2**	**S2A**	B4MOR$^{+/+}$ versus B4MOR$^{-/-}$	FR1<br>3-way ANOVA: Genotype x NosePoke x Time, the latter two as RM variables<br>- Genotype: B4MOR$^{+/+}$ versus B4MOR$^{-/-}$<br>- NosePoke (NP): Active versus Inactive<br>- Time: Test day 1 to 15	- Main effect of Time, $F_{14, 308}$ = 13.95, p < 0.0001<br>- Time x Genotype interaction effect, $F_{14, 308}$ = 0.62, p = 0.85<br>- Main effect of NP, $F_{1, 22}$ = 227.2, p < 0.0001<br>- NP x Genotype interaction effect, $F_{1, 22}$ = 2.16, p = 0.16<br>- NP x Time interaction effect, $F_{14, 308}$ = 22.76, p < 0.0001<br>- Main effect of Genotype, $F_{1, 22}$ = 2.47, p = 0.13<br>- NP x Time x Genotype interaction effect, $F_{14, 308}$ = 0.86, p = 0.6	
			FR5<br>3-way ANOVA: Genotype x NosePoke x Time, the latter two as RM variables<br>- Genotype: B4MOR$^{+/+}$ versus B4MOR$^{-/-}$<br>- NosePoke (NP): Active versus Inactive<br>- Time: Test day 16 to 22	- Main effect of Time, $F_{6, 132}$ = 9.25, p < 0.0001<br>- Time x Genotype interaction effect, $F_{6, 132}$ = 0.72, p = 0.63<br>- Main effect of NP, $F_{1, 22}$ = 435.1, p < 0.0001<br>- NP x Genotype interaction effect, $F_{1, 22}$ = 0.28, p = 0.6<br>- NP x Time interaction effect, $F_{6, 132}$ = 8.38, p < 0.0001<br>- Main effect of Genotype, $F_{1, 22}$ = 0.23, p = 0.64<br>- NP x Time x Genotype interaction effect, $F_{6, 132}$ = 0.52, p = 0.79	
			PR at test day 23<br>2-way ANOVA: Genotype x NosePoke, the latter as a RM variable<br>- Genotype: B4MOR$^{+/+}$ versus B4MOR$^{-/-}$<br>- NosePoke (NP): Active versus Inactive	- Main effect of NP, $F_{1, 22}$ = 74.62, p < 0.0001<br>- Main effect of Genotype, $F_{1, 22}$ = 0.31, p = 0.58<br>- NP x Genotype interaction effect, $F_{1, 22}$ = 0.4, p = 0.53	
	**S2A**	B4MOR$^{+/+}$ versus B4MOR$^{-/-}$	Reversal<br>3-way ANOVA: Genotype x NosePoke x Time, the latter two as RM variables<br>- Genotype: B4MOR$^{+/+}$ versus B4MOR$^{-/-}$<br>- NosePoke (NP): Active versus Inactive<br>- Time: Test day 26 to 28	- Main effect of Time, $F_{2, 44}$ = 38.72, p < 0.0001<br>- Time x Genotype interaction effect, $F_{2, 44}$ = 2.46, p = 0.1<br>- Main effect of NP, $F_{1, 22}$ = 0.06, p = 0.8<br>- NP x Genotype interaction effect, $F_{1, 22}$ = 0.11, p = 0.74<br>- NP x Time interaction effect, $F_{2, 44}$ = 88.39, p < 0.0001<br>- Main effect of Genotype, $F_{1, 22}$ = 0.29, p = 0.6<br>- NP x Time x Genotype interaction effect, $F_{2, 44}$ = 0.77, p = 0.47	
	**S2B**	B4MOR$^{+/+}$ versus B4MOR$^{-/-}$	FR1<br>2-way ANOVA: Genotype x Time, the latter as a RM variable<br>- Genotype: B4MOR$^{+/+}$ versus B4MOR$^{-/-}$<br>- Time: Test day 1 to 15	- Main effect of Time, $F_{14, 308}$ = 45.88, p < 0.0001<br>- Main effect of Genotype, $F_{1, 22}$ = 0.27, p = 0.61<br>- Time x Genotype interaction effect, $F_{14, 308}$ = 0.48, p = 0.94	
			FR5<br>2-way ANOVA: Genotype x Time, the latter as a RM variable<br>- Genotype: B4MOR$^{+/+}$ versus B4MOR$^{-/-}$<br>- Time: Test day 16 to 22	- Main effect of Time, $F_{6, 132}$ = 6.44, p < 0.0001<br>- Main effect of Genotype, $F_{1, 22}$ = 0.32, p = 0.58<br>- Time x Genotype interaction effect, $F_{6, 132}$ = 0.44, p = 0.85	
			PR at test day 23<br>Unpaired t-test	t_{22} = 0.36, p = 0.72	
			Reversal<br>2-way ANOVA: Genotype x Time, the latter as a RM variable<br>- Genotype: B4MOR$^{+/+}$ versus B4MOR$^{-/-}$<br>- Time: Test day 26 to 28	- Main effect of Time, $F_{2, 44}$ = 68.92, p < 0.0001<br>- Main effect of Genotype, $F_{1, 22}$ = 0.78, p = 0.39<br>- Time x Genotype interaction effect, $F_{2, 44}$ = 2.89, p = 0.07	

**Figure S3**	**S3B**	*Gpr151*$^{+/+}$ Females	2-way ANOVAs: Test x Chamber, both as RM variables<br>- Test: 5-10min Habituation bin versus 10-15min social bin<br>- Chamber: Object versus Social	- Main effect of Test, $F_{1, 19} = 95.01$, $p < 0.0001$<br>- Main effect of Chamber, $F_{1, 19} = 1.51$, $p = 0.23$<br>- Test x Chamber interaction effect, $F_{1, 19} = 2.29$, $p = 0.15$	- Habituation versus social test : Object, $p = 0.74$; social, $^{\#}p = 0.045$
	**S3C**	*Gpr151*$^{-/-}$ Females		- Main effect of Test, $F_{1, 14} = 4.73$, $p = 0.047$<br>- Main effect of Chamber, $F_{1, 14} = 1.53$, $p = 0.24$<br>- Test x Chamber interaction effect, $F_{1, 14} = 0.21$, $p = 0.65$	- Habituation versus social test : Object, $p > 0.99$; social, $p = 0.77$
	**S3D**	*Gpr151*$^{+/+}$ Females		- Main effect of Test, $F_{1, 19} = 34.69$, $p < 0.0001$<br>- Main effect of Chamber, $F_{1, 19} = 1.32$, $p = 0.27$<br>- Test x Chamber interaction effect, $F_{1, 19} = 1.32$, $p = 0.27$	- Habituation versus social test : Object, $p = 0.22$; social, $p = 0.99$
	**S3E**	*Gpr151*$^{-/-}$ Females		- Main effect of Test, $F_{1, 14} = 15.86$, $p = 0.001$<br>- Main effect of Chamber, $F_{1, 14} = 0.58$, $p = 0.46$<br>- Test x Chamber interaction effect, $F_{1, 14} = 0.08$, $p = 0.78$	- Habituation versus social test : Object, $p = 0.75$; social, $p = 0.78$
**Figure S4**	**S4B**	Judge for *Oprm1*$^{+/+}$ versus *Oprm1*$^{-/-}$	2-way ANOVA: Test x Chamber, both as RM variables<br>- Test: 5-10min Habituation bin versus 10-15min social bin<br>- Chamber: *Oprm1*$^{+/+}$ versus *Oprm1*$^{-/-}$	- Main effect of Test, $F_{1, 23} = 74.5$, $p < 0.0001$<br>- Main effect of Chamber, $F_{1, 23} = 0.77$, $p = 0.39$<br>- Test x Chamber interaction effect, $F_{1, 23} = 3.21$, $p = 0.087$	- Habituation versus social test: *Oprm1*$^{+/+}$, $^{\#}p = 0.02$; *Oprm1*$^{-/-}$, $p = 0.85$
	**S4C**	Judge for B4MOR$^{+/+}$ versus B4MOR$^{-/-}$	2-way ANOVA: Test x Chamber, both as RM variables<br>- Test: 5-10min Habituation bin versus 10-15min social bin<br>- Chamber: B4MOR$^{+/+}$ versus B4MOR$^{-/-}$	- Main effect of Test, $F_{1, 23} = 65.75$, $p < 0.0001$<br>- Main effect of Chamber, $F_{1, 23} = 1.71$, $p = 0.2$<br>- Test x Chamber interaction effect, $F_{1, 23} = 0.08$, $p = 0.78$	- Habituation versus social test: B4MOR$^{+/+}$, $p = 0.34$; B4MOR$^{-/-}$, $p = 0.34$
	**S4D**	Judge for *Oprm1*$^{+/+}$ versus *Oprm1*$^{-/-}$	2-way ANOVA: Test x Chamber, both as RM variables<br>- Test: 5-10min Habituation bin versus 10-15min social bin<br>- Chamber: *Oprm1*$^{+/+}$ versus *Oprm1*$^{-/-}$	- Main effect of Test, $F_{1, 23} = 21.64$, $p = 0.0001$<br>- Main effect of Chamber, $F_{1, 23} = 0.85$, $p = 0.37$<br>- Test x Chamber interaction effect, $F_{1, 23} = 2.58$, $p = 0.12$	- Habituation versus social test: *Oprm1*$^{+/+}$, $p = 0.55$; *Oprm1*$^{-/-}$, $p = 0.21$
	**S4E**	Judge for B4MOR$^{+/+}$ versus B4MOR$^{-/-}$	2-way ANOVA: Test x Chamber, both as RM variables<br>- Test: 5-10min Habituation bin versus 10-15min social bin<br>- Chamber: B4MOR$^{+/+}$ versus B4MOR$^{-/-}$	- Main effect of Test, $F_{1, 23} = 3$, $p = 0.1$<br>- Main effect of Chamber, $F_{1, 23} = 1.83$, $p = 0.19$<br>- Test x Chamber interaction effect, $F_{1, 23} = 0.05$, $p = 0.83$	
**Figure S5**		B4MOR$^{+/+}$ versus B4MOR$^{-/-}$	2-way ANOVAs: Genotype x Time, the latter as a RM variable<br>- Genotype: B4MOR$^{+/+}$ versus B4MOR$^{-/-}$<br>- Time: 5-min bins	Distance, Time in Center and Time in Corners<br>- Main effect of Genotype, all *P*'s > 0.05<br>- Main effect of Time, $F_{2, 44} = 4.29$, All *P*'s < 0.05<br>- Time x Genotype interaction effect, All *P*'s > 0.05	
**Figure S6**		B4MOR$^{+/+}$ versus B4MOR$^{-/-}$ & *Gpr151*$^{+/+}$ versus *Gpr151*$^{-/-}$	Social preference test<br>Latency to first entry (social chamber)<br>Unpaired t-tests	- B4MOR$^{-/-}$ versus B4MOR$^{+/+}$, $t_{43} = 0.76$, $p = 0.45$<br>- *Gpr151*$^{-/-}$ versus *Gpr151*$^{+/+}$, Males, $t_{37} = 0.37$, $p = 0.71$<br>- *Gpr151*$^{-/-}$ versus *Gpr151*$^{+/+}$, Females, $t_{33} = 0.37$, $p = 0.71$	
**Figure S8**		*Gpr151*-Cre ChR2 versus Controls	2-way ANOVA: Virus x Frequency, the latter as a RM variable	- Main effect of Virus, $F_{1, 24} = 0.55$, $p = 0.78$<br>- Main effect of Frequency, $F_{1, 24} = 3.23$, $p = 0.09$<br>- Virus x Frequency interaction effect, $F_{1, 24} = 0.08$, $p = 0.78$	
