# Supplementary material for: The mu opioid receptor and the orphan receptor GPR151 contribute to social reward in the habenula
Source: Sci Rep. 2022 Nov 24;12:20234. doi: 10.1038/s41598-022-24395-z (PMC9691715; doi:10.1038/s41598-022-24395-z)
Supplement: Supplementary file 2 — Supplementary Figures. [file 41598_2022_24395_MOESM2_ESM.docx]

**The Mu Opioid Receptor and the Orphan Receptor GPR151 Contribute to Social Reward in the Habenula**

**Figure S1. Dendrogram for cluster analysis of social interaction test after chronic social defeat stress or control procedures.** The time spent in the social zone during the social interaction test was used as a measure to allocate the animals to different clusters. Based on this measure, four clusters were drawn with the WARD method. N’s = 7-10/Group.

**Figure S2. B4MOR^-/-^** **mice self-administer a similar number of chocolate-flavored pellets than** B4MOR^+/+^ **mice.** (**A**) The number of active nose-pokes increased over time and was higher than the number of inactive nose-pokes in both B4MOR^+/+^ and B4MOR^-/-^ mice. (**B**) The number of pellets self-administered was also the same in both genotypes with the different ratios tested. FR, Fixed Ratio. PR, progressive Ratio. N’s = 11-13/Group.

**Figure S3. Female *Gpr151^-/-^*** **mice do not express social preference in the three-chamber social preference test.** (**A**) shows a schematic representation of the social preference test. (**B**-**C**) Time spent in the social versus object compartment in *Gpr151^+/+^* and *Gpr151^-/-^* mice, respectively. During the social test, *Gpr151^+/+^* mice increased their time spent in the social compartment. (**D**-**E**) Distance from the social versus object cup in *Gpr151^+/+^* and *Gpr151^-/-^* mice, respectively. ^#^*p* < 0.05, Habituation (Hab) versus Social Test. N’s = 15-20/Group.

**Figure S4. B4MOR^-/-^** **mice do not show atypical social behavior, as do constitutive *Oprm1^-/-^* mice in the “judge” social paradigm.** (**A**) shows a schematic representation of the reverse three chamber social test. (**B**) Time spent by a judge mouse in the *Oprm1^+/+^* and *Oprm1^-/-^* associated compartments. During the social choice test, judge mice increased their time spent in the compartment associated with the *Oprm1^+/+^* mice. (**C**) Time spent by a judge mouse in the B4MOR^+/+^ mice and B4MOR*^-/-^* associated compartments. Judge mice did not make a social choice. (**D**-**E**) Distance from the cups either associated with wild type or mutant mice. ^#^*p* < 0.05, Habituation (Hab) versus Social Test. N’s = 24/Group.

**Figure S5. B4MOR^-/-^ mice and B4MOR*^+/+^*** **mice behave similarly in open field apparatus.** Locomotor activity was the same in both genotypes. The times spent in the center and in the corners of the open field were similar in B4MOR^-/-^ mice and B4MOR*^+/+^* mice suggesting no deficits in anxiety-like phenotype in B4MOR^-/-^ mice. N’s = 12/Group.

**Figure S6.** During the social preference test, the latency to enter the social compartment for the first time was similar in B4MOR^+/+^ and B4MOR^-/-^ mice, as well as in *Gpr151^+/+^* and *Gpr151^-/-^* mice for both males and females. N’s = 15-24/Group.

**Figure S7. DAPI, *Oprm1* and *Gpr151* mRNA in a MHb section from a *Gpr151^+/+^* mouse.** Qualitative observation of MHb cells expressing *Oprm1* mRNA only (yellow arrow), *Gpr151* mRNA only (white arrow) or both (blue arrow).

**Figure S8. Opto-stimulation of MHb-IPN GPR151-positive neurons does not induce avoidance.** *Gpr151*-Cre mice received an injection in the habenula (Hb) of either AAV2.EF1a.DIO.ChR2-mCherry (ChR2, Channelorhodopsin) or AAV2.EF1a.DIO.mCherry (control) and were implanted with an optic fiber (OF) above the interpeduncular nucleus (IPN). ChR2-mice did not avoid the compartment associated with 20-Hz blue-laser photo-stimulation suggesting that GPR151 positive cells stimulation is not aversive. We used the same parameters of stimulation known to promote aversion when habenular MOR-positive cells are stimulated (Bailly et al. 2021). This suggests that the habenular MORs positive cells that promote aversion are not the same than the GPR151 positive neurons. N’s = 13/Group.
