# Supplementary material for: The mu opioid receptor and the orphan receptor GPR151 contribute to social reward in the habenula
Source: Sci Rep. 2022 Nov 24;12:20234. doi: 10.1038/s41598-022-24395-z (PMC9691715; doi:10.1038/s41598-022-24395-z)
Supplement: Supplementary file 3 — Supplementary Information. [file 41598_2022_24395_MOESM3_ESM.docx]

**The Mu Opioid Receptor and the Orphan Receptor GPR151 Contribute to Social Reward in the Habenula**

***Supplementary Methods***

Florence Allain, PhD^1,2^, Michelle Carter, BSc^1^, Sylvie Dumas^3^, Emmanuel Darcq, PhD^1,2^, and Brigitte L. Kieffer, PhD*^1,2^

^1^Douglas Hospital Research Center, Department of Psychiatry, McGill University, Montreal, Canada.

^2^ INSERM U1114, Centre de Recherche en Biomédecine de Strasbourg, Université de Strasbourg, France.

^3^ Oramacell, 75006 Paris, France

***Housing*.** Experimental mice were housed 2-5 per cage under a 12-h light/dark cycle (lights on at 8:00am) in a temperature- and humidity-controlled room. Mice were 2-5 months old at the beginning of testing. Food and water were available *ad libitum* except for the chocolate-pellets self-administration procedure where mice were food restricted 5 days before the start of the experiment and until the end to 85% of their original weight. For each behavioral test, mice were brought in the testing room ~30 minutes before for habituation. To avoid a stress induced by first experimental manipulation, mice were handled for 3 successive days (1-2 min/mouse/day) before the start of an experiment.

***Genotyping PCR*.** Genotyping was done on DNA extracted from ear samples with the respective primers’ sequences for each mouse line:

*Mor^fl/fl^ x Chrnβ4*-Cre [[1](#_ENREF_1)]

Forward Cre primer (GATCTCCGGTATTGA AACTCCAGC)

Reverse Cre primer (GCTAAACATGCTTCATCGTCGG)

Forward myosin gene primer (TTACGTCCATCGTGGACAGC)

Reverse myosin gene primer (TGGGCTGGGTGTTAGCCTTA)

Expected fragment sizes (bp), Cre (595), Myosin (245)

*Oprm1 knock-out* [[2](#_ENREF_2)]

Forward primer (GAGTTAGGAGAATCAGGAGTTCAAG)

Reverse primer (TGCCATGAACATTACGGGCAGAC)

Expected fragment sizes (bp), *Oprm1^-/-^* (422), *Oprm1^+/+^* (648)

*Gpr151 knock-out*

The *Gpr151-eGFP* knock-in mutant mouse line was established at the Institut Clinique de la Souris, Strasbourg France (PHENOMIN, <http://www.phenomin.fr>). The targeting vector was constructed as follows. A 3 kb fragment encompassing *Gpr151* the 5’UTR present in exon 1 and the promoter and regulatory sequence of *Gpr151* as well as the eGFP (717 bps) were amplified by PCR (from RP23-10B2 for the genomic DNA sequence and an eGFP containing plasmid respectively) and subcloned by SLIC (Sequence and Ligation-Independent) cloning in an ICS proprietary vector. This ICS vector has a floxed Neomycin resistance cassette with an autoexcision transgene. A 3’ homology arm of 2.3 kb encompassing the 3’ UTR and the 3’ genomic sequence was amplified by PCR and subcloned in step1 plasmid to generate the final targeting construct. The linearized construct was electroporated in C57BL/6N mouse embryonic stem (ES) cells (ICS proprietary line). After G418 selection, targeted clones were identified by long-range PCR and further confirmed by Southern blot with an internal (Neo) probe and a 5’ external probe. One positive ES clone was validated by karyotype spreading and microinjected into BALB/C blastocysts. Resulting male chimeras were bred with wild type females. Germline transmission with the direct excision of the selection cassette was achieved in the first litter.

The *Gpr151* *knock-out* mice genotyping was done in Charles River, France.

Forward primer (GGAGGGTCCAGAGGTGTCTTC)

Reverse primer (GTGAACAGCTCCTCGCCCTTG)

Expected fragment sizes (bp), *Gpr151^-/-^* (123), *Gpr151^+/+^* (276)

*Gpr151*-Cre

*Gpr151*-Cre mouse line was developed by [[3](#_ENREF_3)]. Forward and reverse Cre primers were the same than for the *Oprm1^l/fl^ x Chrnb4*-Cre mouse line.

***Fluorescent In Situ Hybridization (FISH).*** FISH was performed as described previously [[4](#_ENREF_4)]. Antisense riboprobe for the detection of *Gpr151* mRNA: NM_181543.2 sequence 360-1244 was designed to recognize the coding sequence of the *Gpr151* mRNA. The riboprobe was synthesized with digoxigenin-labeled ribonucleoside tri-phosphate. Cryosections were air-dried, fixed in 4% paraformaldehyde and acetylated in 0.25% acetic anhydride/100 mM triethanolamine (pH 8). Sections were hybridized for 18 h at 65°C in 100 µl of formamide-buffer containing 1 µg/ml digoxigenin-labeled *Gpr151* riboprobe. Sections were washed at 65°C with SSC buffers of decreasing strength and blocked with 20% fetal bovine serum and 1% blocking solution. Fluorescein epitopes were detected with horseradish peroxidase (HRP) conjugated anti-digoxigenin antibody at 1:1000 and revealed using Cy2-tyramide at 1:100. Nuclear staining was performed with 4' 6-diamidino-2-phenylindole (DAPI). All slides were scanned on a 20x resolution NanoZoomer 2.0-HT (Hamamatsu, Japan). Image analysis was performed using the ndp2.view software (Hamamatsu).

***Immunohistochemistry.*** *Gpr151^+/+^* and *Gpr151^-/-^* mice were anesthetized with an intraperitoneal injection of a mix of Ketamine/Xylazine (K: 125mg/kg – 100mg/ml; X: 10mg/kg – 20mg/ml). When the absence of reflexes was noticed, mice were perfused (intracardiac) with Phosphate Buffer Saline 1X first (~2min, Fisher Scientific) followed by 4% paraformaldehyde (~5min, Fisher Scientific) at a rate of 10ml of the corresponding solution per minute. Brains were then collected and postfixed for 24 hours in a 4% paraformaldehyde solution and for 48 supplemental hours in a 30% solution of sucrose (Fisher Scientific) before being conserved at -80°C. 30-µm coronal brain slices with a 50-60° angle were made with a cryostat (Epredia CryoStar NX70, Fisher Scientific). Brain sections were treated for immunohistochemistry following this order: 3 x 10-min in PBS-T (Triton 100X 0.1%, Sigma-Aldrich), 1 hour in blocking buffer (PBS, Normal Goat Serum 3% (Fisher Scientific), Triton 100X 0.2%), overnight in blocking buffer with primary antibody (Rabbit anti-Green Fluorescent Protein 1:2000, A11122, ThermoFisher Scientific), 3 x 10-min in PBS-T, 2 hours in blocking buffer with secondary antibody (Goat anti-Rabbit a647 1:2000, A21244, ThermoFisher Scientific), 3 x 10-min in PBS-T, 1 x 10-min in 0.5 mg/ml DAPI (RRID:AB_2307445, ThermoFisher Scientific) and final in PBS-1X before being mounted on SuperFrost Plus Adhesion Microscope Slides (J1800AMNZ, Epredia) using a Mowiol mounting medium. For fluorescent microscopy, images were scanned using a widefield Zeiss Apotome microscope, objective 20X.

***Chronic social defeat stress (CSDS) and social interaction test (SIT)*.** B4MOR^+/+^ and B4MOR*^-/-^* mice were either exposed to a CSDS procedure (defeated mice) or a control procedure (control mice) for 10 days as previously described in [[5](#_ENREF_5),[6](#_ENREF_6)]. CD1 mice (for the CSDS procedure) or c57bl6:Sv129 mice (for the control procedure) were housed on one side of 2-compartments rat cages separated by transparent dividers composed with holes to allow sensorial communication (olfactory, visual and auditory stimuli). Defeated B4MOR^+/+^ and B4MOR*^-/-^* mice were placed in direct contact with an aggressive CD1 mouse for a maximum of 5 minutes or 10 attacks per day. Then, they were transferred to the other side of the 2-compartments rat cage for the next 24 hours where they were protected from CD1 attacks but still received continuous passive exposition to the aggressor. This procedure was repeated 10 days, and experimental mice were exposed to a different CD1 aggressor every day of the procedure. To parallel the CSDS procedure, controls *B4M* B4MOR^+/+^ and B4MOR*^-/-^* mice were transferred from one cage to another every day to be in indirect contact with different c57bl6:Sv129 mice (no direct contacts in controls). At the end of the last session of CSDS/control procedure, mice were housed 1/cage in regular mouse cages. 24 hours after the end of the procedure, a social interaction test was done to evaluate the development of a depressive-like state in defeated mice [[5](#_ENREF_5),[6](#_ENREF_6)]. In this test, mice were allowed to freely explore an open field apparatus (45 cm^2^, 50 cm height) where a wire mesh enclosure (11 x 8 cm, with mesh part, 12.5 x 9 cm, 42 cm height) was placed against one wall (**Fig. 1A**). A social interaction zone was drawn around the wire mesh enclosure (13.5 x 25.5 cm) and the time spent in this zone served as a measure of social interaction (gray zone in **Fig. 1A**). The wire mesh enclosure was empty for the first 150 seconds of the test (habituation) and contained a novel CD1 mouse (that has never been in contact with an experimental mouse before) for the next 150 seconds of the test (SIT). The time spent in the social zone was compared with the time spent in the four corners of the box. For the hierarchical clustering analysis, individual mice were grouped together based on similar amount of time spent in the CD1 zone. The clustering algorithm used was the WARD method and the squared Euclidian distance was the measure of similarity. The dendrogram revealed four distinct clusters (**Fig. S1**).

***Social preference test.*** Mice were exposed to a 3-compartments handmade box (total 58 cm x 22 cm; 22cm^2^ for each social-paired and object-paired compartment separated by a 9.5 cm x 22 cm central compartment; A closing mechanism allowed to individualize each compartment; Indirectly lit at ~60 Lux; **Fig. 2A**) for 10 minutes with two empty cups (bottom Ø 10 cm, top Ø 7 cm) for habituation and for five supplemental minutes with an interactor placed under a cup (c57bl6:Sv129WT interactors for B4MOR^+/+^ and B4MOR*^-/-^* mice or c57bl6WT interactors for *Gpr151^+/+^* and *151^-/-^* mice were previously habituated to be confined under the cups). During social test, an empty cup remained in the non-social/object-paired compartment. The compartment associated with social reward was counterbalanced between conditions. Times spent in the social zone, object zone and the distance from respective cups were monitored during habituation and social test. The last 5-min bin of the habituation phase was compared to the 5-min social test. An increased time spent in the social-paired compartment was used as an index of social novelty preference/sociability [[7](#_ENREF_7),[8](#_ENREF_8)]. Female c57bl6WT interactors were used to test social preference in females *Gpr151^+/+^* and *151^-/-^* [[9](#_ENREF_9)].

***Social conditioned place preference (CPP) test*.** B4MOR^+/+^ and B4MOR*^-/-^* mice were allowed to freely explore a 3 chambers apparatus (iMetronic, France) for 15 minutes on day 1 (pre-test). In this apparatus, two compartments (22 x 22 cm, each h25 cm) were separated by a central compartment (22 x 8 cm). The two compartments were distinct by their color, floor texture and spatial configuration (**Fig. 3A**). At the end of the day, mice were socially isolated and housed 1/cage. From day 2 to day 9 (8 days of social conditioning), mice were confined 30 minutes alone in one compartment every morning and confined with their previous cage-mates 30 minutes in the other compartment every afternoon (adapted from [[1](#_ENREF_1),[10](#_ENREF_10)]). Social- and non-social-paired compartments were allocated based on the time spent in each compartment on day 1 to promote an unbiased social conditioning with an initial preference of ~50%. At the end of social conditioning, mice were again allowed to freely explore the 3-chambers apparatus for 15 minutes on days 10, 14 and 18 (post-tests). A CPP score was calculated as the percentage of time spent in the social-paired compartment during the post-test minus the same percentage during the pre-test. Each compartment was equipped with 4 infrared photobeams. The measure of beam ‘breaks’ and ‘release’ (e.g. crossing forward the 4 photobeams in one compartment is equivalent for mice to make 8 beam ‘breaks’ and ‘release’) was used as an index of locomotor activity during the tests.

***Operant self-administration of food pellets*.** B4MOR^+/+^ and B4MOR*^-/-^* mice were allowed to self-administer 45-mg chocolate-flavored pellets in operant boxes equipped with two ports (iMetronic, France). Nose-pokes (NP) in the active port were reinforced by food delivery in a food receptacle (located in the middle of the two ports) and NP in the inactive port had no programmed consequences (**Fig. S2**). The protocol was adapted from [[11](#_ENREF_11)]. A house light and a fan turned on at the beginning of the session and turned off at the end. The cue-light above the active port turned on to indicate food availability. When the mouse reached the fixed-ratio (FR) required to obtain one food pellet (FR1 or FR5, 1NP or 5NP, respectively), the cue-light turned off until the mouse went in the food receptacle to collect the food pellet. Then, the cue-light turned on again. Mice received daily sessions in this order: Fifteen FR1-sessions, seven FR5-sessions, one Progressive Ratio (PR) session, two FR1-sessions, three FR1-sessions where the two nose-pokes were reversed (to test flexible responding on a reversal task [[12](#_ENREF_12)]) and a last regular FR1-session. FR1- and FR5-sessions lasted 20 minutes each except for the first three FR1-sessions that lasted 30 minutes each to improve the learning process. Under PR, the number of NP in the active port required to obtain the next chocolate pellet was multiplied by two and the cue light above the active port turned off for 1 second every time a mouse reached the next ratio. A PR-session ended if the mouse did not make any NP in the active port for 10 minutes.

***Reverse three chamber social test*.** C57bl6:Sv129WT “judge” mice were allowed to freely explore a 3-compartments handmade box (as described previously for the social preference test) for 10 minutes with empty cups in the two opposite compartments for habituation. Then, for the subsequent 10-min social choice test, judge mice were exposed to either the combination *Oprm1*^+/+^ versus *Oprm1*^-/-^ mice (one under each cup) or the combination B4MOR^+/+^ and B4MOR*^-/-^* mice (**Fig. S5A**). *Oprm1*^+/+^ and *Oprm1*^-/-^ pairs or B4MOR^+/+^ and B4MOR*^-/-^* pairs were habituated to cups’ confinement beforehand. Compartments were counterbalanced within each combination. “Judge” mice first exposed to *Oprm1*^+/+^ and *Oprm1*^-/-^ pairs and were next tested for B4MOR^+/+^ and B4MOR*^-/-^* pairs and vice versa (50/50). Times spent in the compartments and the distance from respective cups were monitored during habituation and social choice test. An increased time spent in the *Oprm1*^+/+^ or B4MOR^+/+^ paired compartment was used as a judgment of “atypical” *Oprm1*^-/-^ or B4MOR*^-/-^* mice social behaviour, respectively [[13](#_ENREF_13),[14](#_ENREF_14)].

***Open Field.*** B4MOR^+/+^ and B4MOR*^-/-^* mice were placed in an open-field arena (in cm, L41 x l41 x h31) for 15 minutes indirectly lit at ~150 Lux (**Fig. S6**). Locomotor- and anxiety-like behaviors [time spent in the center zone (20 cm^2^) versus in the corners (10 cm^2^ each)] were measured.

***Real Time Place Testing in Gpr151-Cre mice.*** Anaesthetized (isoflurane, 5% for induction, 2-3% for maintenance) *Gpr151*-Cre mice received a unilateral injection of either AAV2.EF1a.DIO.ChR2-mCherry (RRID: Addgene_20297) or AAV2.EF1a.DIO.mCherry in the habenula (0°, AP: -1.34, ML: -0.25, DV: -2.75, 300-nl injection) and were implanted with an optic fiber above the interpeduncular nucleus (10° ML angle, AP: -3.12, ML: -0.9, DV: -4.55). After recovery of the surgery, mice were first habituated to be connected to a fiber-optic rotary joint linked to a laser diode fiber light source (Doric). Next, mice were laser-connected and placed in an apparatus divided in two identical chambers (**Fig. S8**). Mice had free access to the two compartments for 20 minutes but only one compartment was associated with blue-laser (473 nm) photo-stimulation. Mice systematically started the session in the designed non-paired photo-stimulation side of the apparatus and this side was counterbalanced between subjects. Mice were tested at a 0-Hz frequency one day and at a 20-Hz photo-stimulation frequency the second day (10-ms pulse width, 50-ms pulse frequency). The time spent in the stimulation side between ChR2 and Controls mice at 0 and 20Hz was measured and served as an index of real time place preference or avoidance in case of increased or decreased time in the 20-Hz associated chamber for the ChR2 group, respectively. In the end of the experiment, histology was made to include only mice with virus-infected habenula and visible optic fiber tract above the IPN (example in **Fig. S8**).

***Data analysis.*** Poly-files software (iMetronic) was used to extract data from social CPP and operant self-administration of food pellets experiments. For other tests, mice behavior was recorded using a CDD camera connected to an Anymaze software. All data were next transferred to excel data sheets and analyzed using Prism.

**REFERENCES**

1 Boulos LJ, Ben Hamida S, Bailly J, Maitra M, Ehrlich AT, Gaveriaux-Ruff C, et al. Mu opioid receptors in the medial habenula contribute to naloxone aversion. Neuropsychopharmacology. 2020;45(2):247-55.

2 Matthes HW, Maldonado R, Simonin F, Valverde O, Slowe S, Kitchen I, et al. Loss of morphine-induced analgesia, reward effect and withdrawal symptoms in mice lacking the mu-opioid-receptor gene. Nature. 1996;383(6603):819-23.

3 Kobayashi Y, Sano Y, Vannoni E, Goto H, Suzuki H, Oba A, et al. Genetic dissection of medial habenula-interpeduncular nucleus pathway function in mice. Front Behav Neurosci. 2013;7:17.

4 Dumas S, Wallén-Mackenzie Å. Developmental Co-expression of Vglut2 and Nurr1 in a Mes-Di-Encephalic Continuum Preceeds Dopamine and Glutamate Neuron Specification. Frontiers in cell and developmental biology. 2019;7:307.

5 Golden SA, Covington HE, 3rd, Berton O, Russo SJ. A standardized protocol for repeated social defeat stress in mice. Nat Protoc. 2011;6(8):1183-91.

6 Torres-Berrio A, Lopez JP, Bagot RC, Nouel D, Dal Bo G, Cuesta S, et al. DCC Confers Susceptibility to Depression-like Behaviors in Humans and Mice and Is Regulated by miR-218. Biol Psychiatry. 2017;81(4):306-15.

7 Nadler JJ, Moy SS, Dold G, Trang D, Simmons N, Perez A, et al. Automated apparatus for quantitation of social approach behaviors in mice. Genes Brain Behav. 2004;3(5):303-14.

8 Becker JA, Clesse D, Spiegelhalter C, Schwab Y, Le Merrer J, Kieffer BL. Autistic-like syndrome in mu opioid receptor null mice is relieved by facilitated mGluR4 activity. Neuropsychopharmacology. 2014;39(9):2049-60.

9 Rein B, Ma K, Yan Z. A standardized social preference protocol for measuring social deficits in mouse models of autism. Nat Protoc. 2020;15(10):3464-77.

10 Trezza V, Damsteegt R, Achterberg EJ, Vanderschuren LJ. Nucleus accumbens mu-opioid receptors mediate social reward. J Neurosci. 2011;31(17):6362-70.

11 Ben Hamida S, Mendonca-Netto S, Arefin TM, Nasseef MT, Boulos LJ, McNicholas M, et al. Increased Alcohol Seeking in Mice Lacking Gpr88 Involves Dysfunctional Mesocorticolimbic Networks. Biol Psychiatry. 2018;84(3):202-12.

12 Laughlin RE, Grant TL, Williams RW, Jentsch JD. Genetic dissection of behavioral flexibility: reversal learning in mice. Biol Psychiatry. 2011;69(11):1109-16.

13 Shah CR, Forsberg CG, Kang JQ, Veenstra-VanderWeele J. Letting a typical mouse judge whether mouse social interactions are atypical. Autism Res. 2013;6(3):212-20.

14 Toddes C, Lefevre EM, Brandner DD, Zugschwert L, Rothwell PE. Mu Opioid Receptor (Oprm1) Copy Number Influences Nucleus Accumbens Microcircuitry and Reciprocal Social Behaviors. J Neurosci. 2021.
